# Repurposing picropodophyllin as a potential thyroid eye disease treatment via delaying mitotic clonal expansion through a patient‐derived preclinical platform

**DOI:** 10.1002/ctm2.1218

**Published:** 2023-04-03

**Authors:** Jing Hu, Jiali Wu, Qihuang Jin, Ling Zhang, Ning Shen, Yiyang Shu, Lu Cheng, Jian Zhang, Guangyi Hu, Kangjia Lv, Qizhi Jian, Hui Chen, Fang Zhang, Xiaodong Sun

**Affiliations:** ^1^ National Clinical Research Center for Eye Diseases Shanghai General Hospital Shanghai Jiao Tong University School of Medicine Shanghai China; ^2^ Shanghai Key Laboratory of Fundus Diseases Shanghai China; ^3^ Shanghai Engineering Center for Visual Science and Photomedicine Shanghai China; ^4^ Shanghai Engineering Center for Precise Diagnosis and Treatment of Eye Diseases Shanghai China; ^5^ School of Medicine University of Electronic Science and Technology of China Chengdu Sichuan Province China; ^6^ Eye School of Chengdu University of Traditional Chinese Medicine Chengdu Sichuan Province China; ^7^ University of Shanghai for Science and Technology Shanghai China

Dear Editor,

Thyroid eye disease (TED), the most frequent orbital disease and the leading cause of proptosis in adults, is a debilitating, disfiguring, and even sight‐threatening disease.^1^ Drugs as alternative options are required for TED patients who need surgery to control orbital compression. Targeting the enhanced adipogenesis of orbital tissue removed by surgery has therapeutic potential for TED by inhibiting the adipogenesis of orbital fibroblasts (OFs). We have developed lenvatinib as a novel therapeutic option for TED based on the primary OFs in‐vitro model.^2^ However, translational research is hampered by the limited number of OFs and the lack of reproducible preclinical platforms for drug discovery.^3^ Thus, we generated a TED patient‐derived orbital fibroblast cell line (OF‐CL) with proliferative and adipogenic capacity, which was further applied in high‐throughput drug screening systems, 2D and 3D in vitro models, and xenograft in vivo models for drug repurposing in TED without a dramatic loss of inherited genotypes. Taking advantage of drug repurposing, we identified picropodophyllin (PPP), recognized as an inhibitor of insulin‐like growth factor‐1 receptor (IGF‐1R) and in clinical phase II in lung cancer, with anti‐adipogenic therapeutic potential for TED.

The OF‐CL was established through immortalisation of OFs that were isolated from the orbital adipose tissue of TED patients (Figures S1 and 1A). It possessed a fibroblast‐like morphology and the enhanced proliferative capacity (Figure 1B,C). RNA‐sequencing analysis indicated proliferation characteristics at the transcriptomic level (Figures 1D,E and S2A,B). We also evaluated the adipogenesis capacity of OF‐CL and OFs (Figure 1F). Brightfield images and Bodipy staining showed similar formations of intracellular lipid droplets during adipogenic differentiation (Figures 1G,H and S2C), which were confirmed by RNA‐sequencing analysis and qPCR test with genes involved in the formation of mature adipocytes and lipid synthesis including (*C/EBP*)*α*, fatty acid‐binding protein 4 (*FABP4*), perilipin 1 (*PLIN1*), peroxisome proliferator‐activated receptor gamma (*PPARγ*) and adiponectin (*ADIPOQ*) (Figure S2D–F).^2^

**FIGURE 1:**
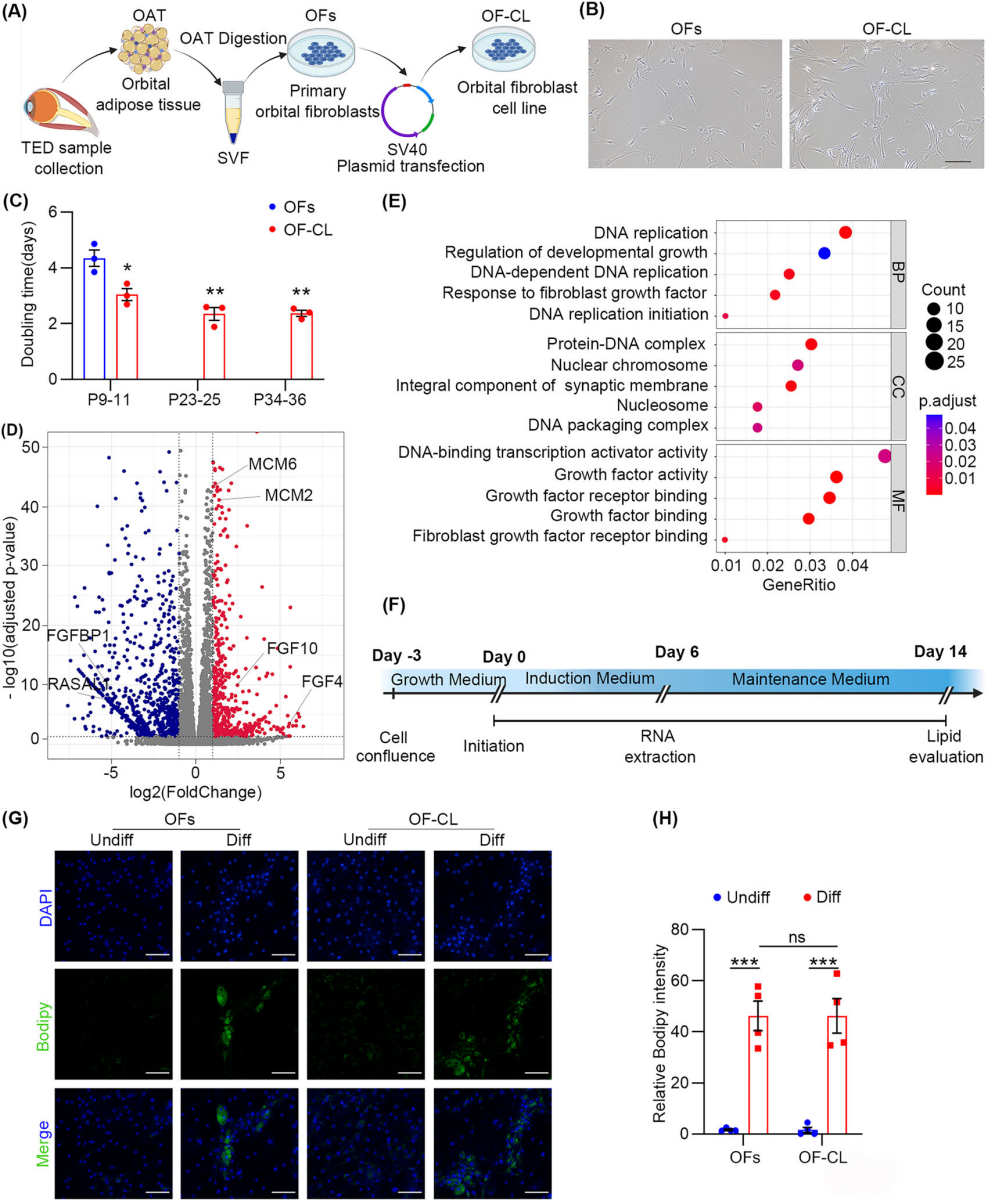
Generation and assessment of a human orbital fibroblast cell line (OF‐CL) from a patient with thyroid eye disease (TED). (A) Schematic view of the construction of an orbital adipose tissue (OAT)‐derived OF‐CL from a TED patient. The stromal vascular fraction (SVF) was isolated from the OAT of a 35‐year‐old female TED patient who underwent decompression surgery, and cultured in dishes for primary orbital fibroblasts (OFs). SV40 large T antigen was transformed into OFs using a retroviral system to generate the immortalised OF‐CLs. (B) Brightfield microscopy photo of OFs and OF‐CL (bar = 200 μm). (C) Doubling time of different generations of OFs and OF‐CL (^*^ *p* < .05, ^**^ *p* < .01). (D) Volcano plot showed differentially expressed genes (DEGs) between OFs and OF‐CL with absolute fold change ≥2 and false discovery rate <0.05 (*n* = 3 for each cell type). Blue and red dots indicate upregulated DEGs in the OF and OF‐CL groups, respectively. All other genes are labelled as grey dots. (E) Gene Ontology (GO) analysis of DEGs showed that proliferation‐related signalling pathways were enriched in OF‐CL. (F) Schematic timeline of adipogenic differentiation and assessment in the 2D culture of OFs or OF‐CL. A total of 8 × 10^3^ cells were plated into each well of 96‐well plates, and grew to 100% confluence in growth medium on day −3. Cells were cultured for another 3 days in normal growth conditions before induction medium was employed on day 0, and the medium was refreshed every 3 days. The medium was changed into the maintenance medium on day 6 and refreshed every three days. RNA extraction was conducted on the day 6 for the detection of adipogenic differentiation marker genes. An assessment of lipid accumulation was performed on day 14. (G) Bodipy and 4',6‐diamidino‐2‐phenylindole (DAPI) staining indicated the intracellular lipid droplets of adipocytes on day 14 (bar = 50 μm). (H) Quantitative analysis of relative Bodipy intensity (^***^ *p* < .001).

Drug repurposing is a safe and successful strategy to speed up the drug discovery and development processes for existing drugs.^4^ To identify candidates for drug repurposing in TED adipogenesis upon patient‐derived OF‐CL, we first hand‐curated a collection of 153 small molecule compounds targeting genes involved in adipogenesis pathways of white adipose tissue from registered clinical trials (Table S3).^5^ To speed up the screening, we set up a quantitative high‐throughput screening system based on patient‐derived OF‐CL to test the rate of anti‐adipogenesis of these 153 compounds via Bodipy staining (Figure 2A). As shown in Figure 2B, although there were no beneficial effects on the anti‐adipogenesis of TED by several compounds that have been documented to inhibit adipogenesis in mesenteric adipose tissue, such as resveratrol and genistein. We identified 25 candidates with anti‐adipogenic therapeutic potential for TED (Table S1). The main documented targets of these compounds was defined as vascular endothelial growth factor receptor (VEGFR), c‐Kit, IGF‐1R, mammalian Target of Rapamycin (mTOR), p38 mitogen‐activated protein kinase (p38 MAPK), COX, etc.

**FIGURE 2:**
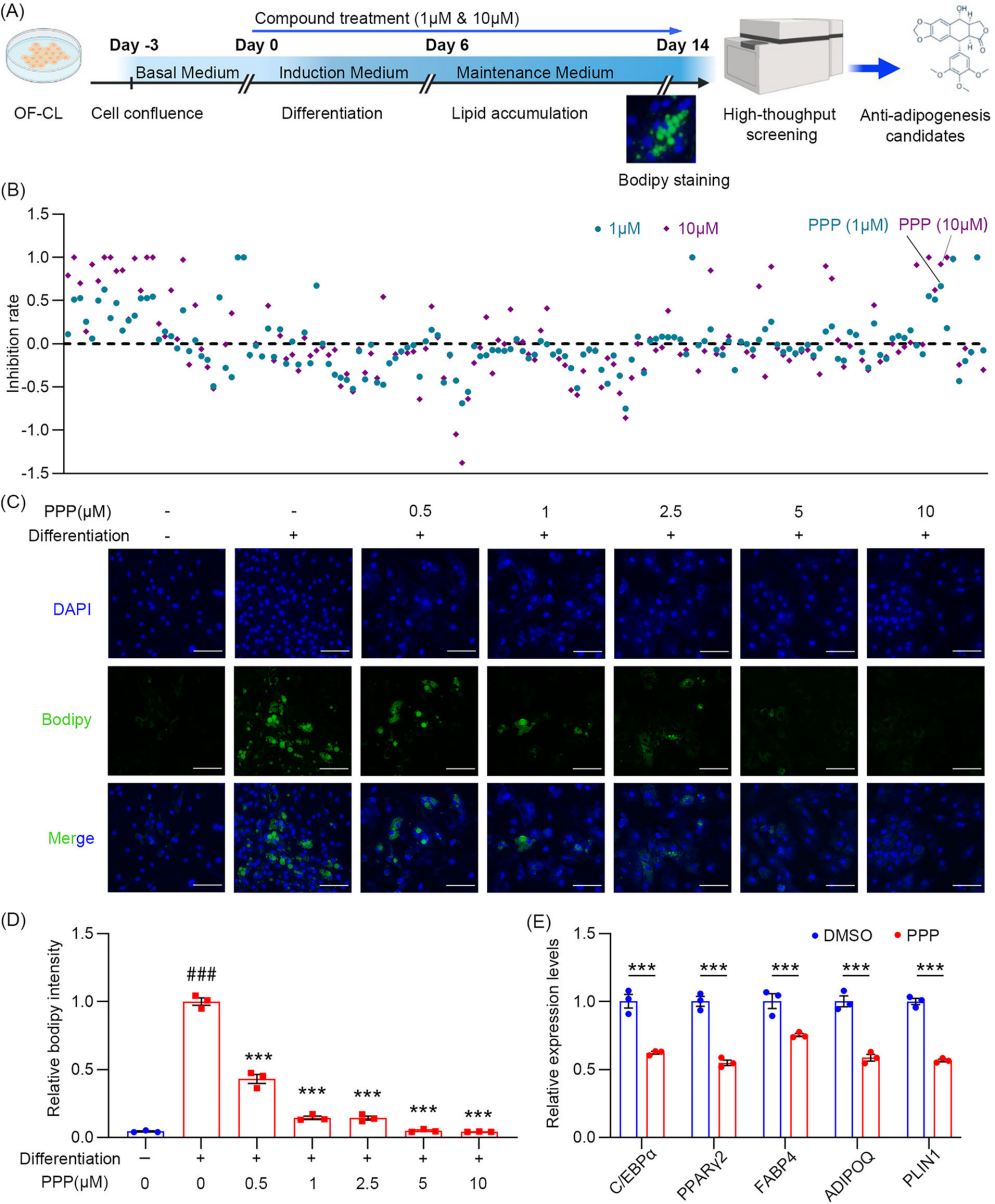
Picropodophyllin (PPP) isolated from high‐throughput screening shows anti‐adipogenesis potentials to thyroid eye disease (TED) upon orbital fibroblast cell line (OF‐CL) and orbital fibroblast (OF) differentiation. (A) Strategy of the high‐throughput screening for small molecules with anti‐adipogenesis potential based on patient‐derived OF‐CL. After 14 days of adipogenic differentiation treated with each compound at indicated concentration, Bodipy and DAPI staining were conducted to quantitatively evaluate the amount of lipid accumulation in each well. (B) Scatter plot of inhibition rates of adipogenesis was calculated through Bodipy intensity for each compound assessed in the high‐throughput screening upon OF‐CL differentiation. Green and purple dots represent 1 and 10 μM concentration groups, respectively (*n* =  2 for each treatment). (C) With the increase in PPP concentration, the relative Bodipy intensity of differentiated OFs decreased on day 14 after adipogenesis induction (bar = 50 μm). (D) The quantitative analysis of relative Bodipy intensity is shown (^###^ *p* < .001 vs. undifferentiated control, ^***^ *p* < .001 vs. differentiated control). (E) The mRNA expression levels of adipogenesis marker genes were detected by RT‐qPCR analysis (^***^ *p* < .001 vs. differentiated control).

PPP among 25 candidates is in first and second phase clinical trials of lung cancer^6^ and shares the IGF‐1R target with teprotumumab antibody, which is the first and only Food and Drug Administration‐approved drug of TED.^7^ To validate the in vitro inhibitory effect of PPP on adipogenesis of TED, we investigated the anti‐adipogenesis effect of PPP on primary OFs. It showed a dose‐dependent decrease in intracellular lipid accumulation and adipogenesis‐related mRNA expression (Figure 2C–E).

To explore the anti‐adipogenesis mechanisms of PPP, we investigated the adipogenesis stages disturbed by PPP (Figure 3A).^8^ PPP suppressed expression of *C/EBPδ* and *C/EBPβ* in the early stage, C/EBPα and PPARγ in the intermediate stage and FABP4 and ADIPOQ in the late stage (Figures 3B and S4). During early stage of differentiation, preadipocytes were reported to undergo mitotic clonal expansion (MCE) with about two rounds of division to proliferation.^9^ *C/EBPδ* and *C/EBPβ* are the core transcription factors of MCE. To investigate whether PPP affects early proliferation, we observed a reduction in cell number in the PPP‐treated group at 48 and 96 h (Figure 3C) and a decrease in proliferation indicated by EdU‐positive cells (Figure 3D,E). The MCE regulatory genes, including cyclin‐dependent kinase (*CDK*)4, *CDK6* and *Cyclin D1*, in the first 24 h of adipogenesis were also suppressed by PPP at transcriptional level (Figure S5A).

**FIGURE 3:**
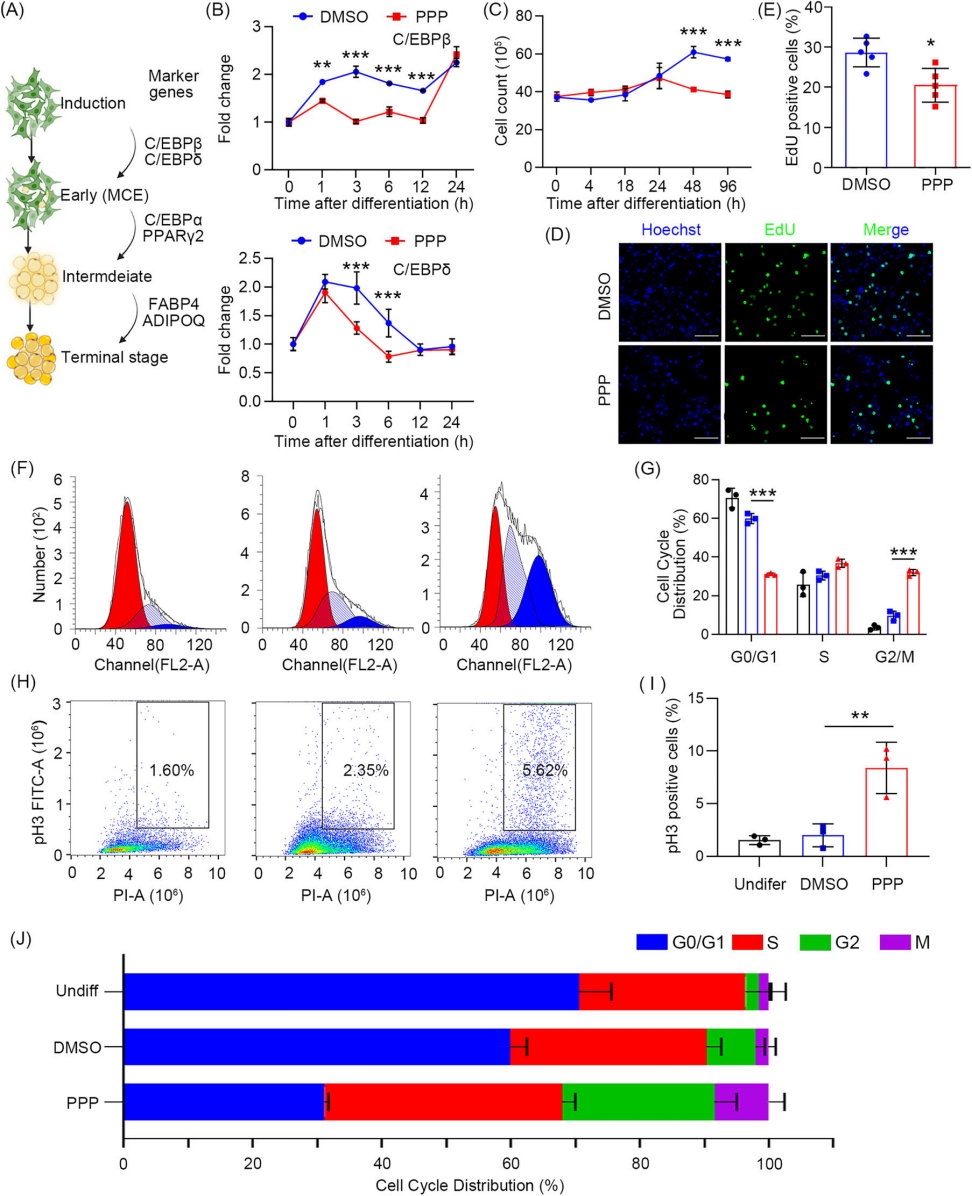
Picropodophyllin (PPP) inhibits adipogenesis by affecting the mitotic clonal expansion in early differentiation. (A) Schematic representation of different stages during adipogenic differentiation. (B) RT‐qPCR analysis of C/EBPβ and C/EBPδ after adipogenic induction with dimethylsulfoxide (DMSO) or 1 μM PPP treatment (^**^ *p* < .01, ^***^ *p* < .001). (C) Cell numbers at 0, 4, 18, 24, 48 and 96 h after adipogenic induction treated with DMSO or 1 μM PPP (^***^ *p* < .001). (D) Representative images of EdU staining in differentiated orbital fibroblast cell line (OF‐CL) treated with DMSO or 1 μM PPP for 48 h (bar = 50 μm). (E) Quantification of the ratio of EdU‐positive cells to total Hoechst‐positive cells (^*^ *p* < .05). (F) Flow cytometry analysis of cell cycle distribution in DMSO‐ and PPP‐treated groups at 24 h after adipogenic differentiation. Cell cycle distribution was determined by flow cytometry using propidium iodide staining. (G) The percentages of OF‐CL treated with induction medium supplemented with 1 μM PPP or DMSO for 24 h at different cell cycle phases were analysed by ModFit software (^*^ *p* < .05, ^***^ *p* < .001). (H) Flow cytometry analysis of p‐histone H3‐positive cells in DMSO‐ and PPP‐treated groups at 16 or 24 h after adipogenic differentiation. (I) The percentages of p‐histone H3‐positive cells treated with induction medium supplemented with 1 μM PPP or DMSO for 24 h were analysed by FlowJo software (^**^ *p* < .01, ^***^ *p* < .001). (J) Percentage of cells in G0/G1, S, G2 and M phases after treatment with DMSO or PPP.

To further determine the specific phases of the cell cycle blocked by PPP during MCE, ploidy analysis was performed to analyse the cell distribution at 24 and 16 h after differentiation initiation. Phosphorylate histone H3, M phase cell marker, was used to distinguish cells in G2 phase or M phase (Figures 3F–I and S5B–F). These data suggest that PPP may cause G2/M arrest and inhibit the MCE process (Figure 3J).

Three‐dimensional culture models provide an alternative to 2D cell culture to closely mimic the cellular microenvironment in diseases.^10^ To assess the anti‐adipogenesis function in 3D culture systems, we employed sodium carboxymethyl cellulose and Matrigel to form a fibroblast spheroid (Figure 4A,B). After 21‐day adipogenic differentiation, PPP suppressed 68.31% of lipid accumulation in spheroids of OFs compared with control (Figure 4C,D). The mRNA levels of adipogenic marker genes were significantly reduced in the PPP group (Figure S6A).

**FIGURE 4:**
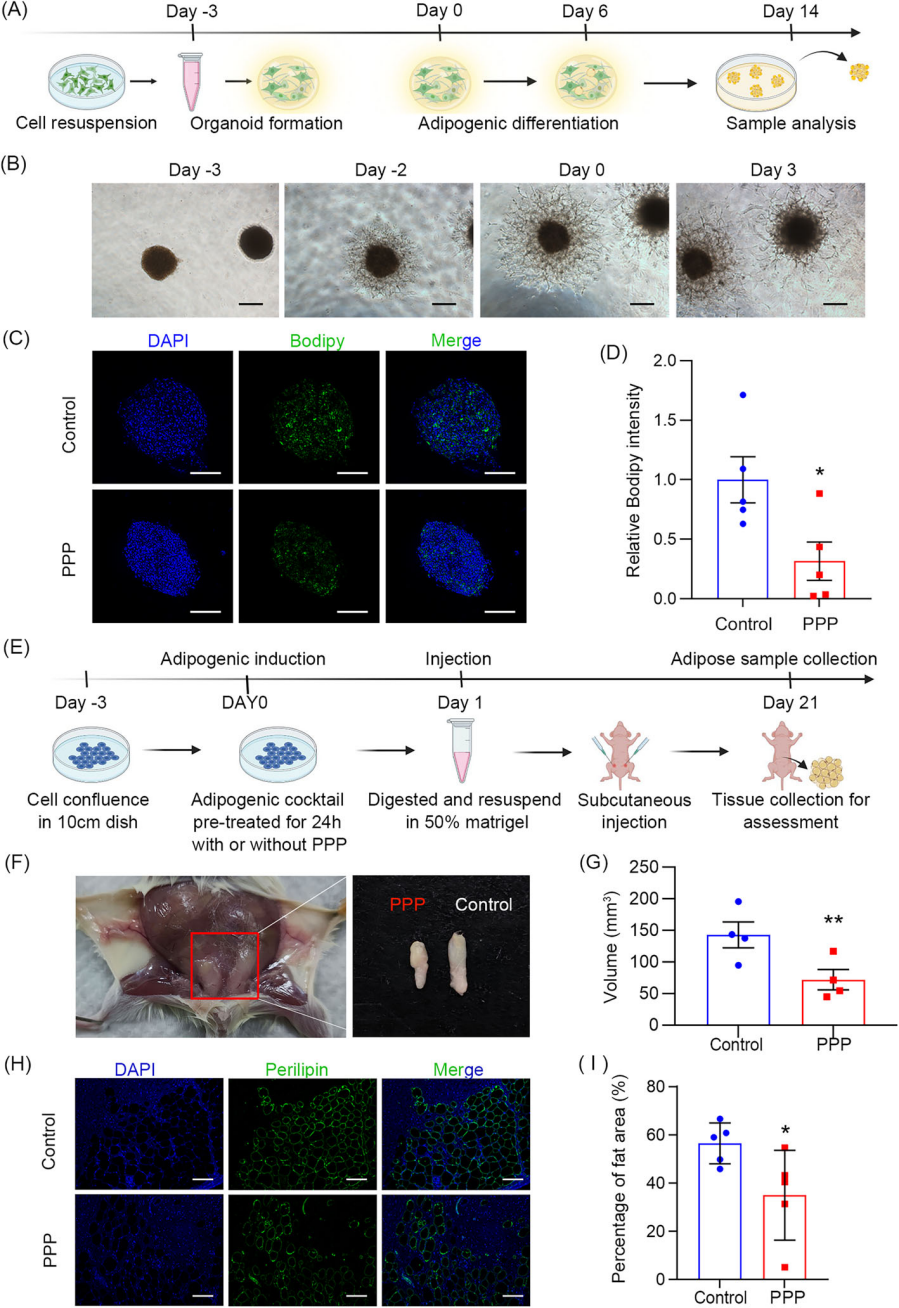
Picropodophyllin (PPP) attenuates adipogenesis in 3D cell culture model and mammalian fat depots of immunodeficient mouse implantation model. (A) Schematic depicting the construction of 3D cell culture model formed by orbital fibroblasts (OFs) with Matrigel. A total of 2 × 10^6^ cells were resuspended in 1 mL growth medium, and a medium drop of 25 μL was placed under the lid for spheroid formation. After being transferred into Matrigel followed by adipogenic differentiation, spheroids were collected and evaluated on day 14. (B) Brightfield images of OFs 3D spheroids on days −3, 0 and 3 (bar = 200 μm). (C) Representative images of DAPI and Bodipy staining 3D spheroids on day 14 (bar = 250 μm). (D) Quantitative analysis of the relative Bodipy fluorescence intensity in 3D spheroids of two groups (^**^ *p* < .01). (E) Schematic depicting the construction of orbital fibroblast cell line (OF‐CL) adipogenesis model in vivo. A total of 2 × 10^6^ OF‐CLs pre‐treated with induction medium supplemented with DMSO or PPP for 24 h were implanted into the left and right inguinal areas of immunodeficient mice, respectively. Mice were sacrificed on day 21 and the OF‐CL/Matrigel plugs were harvested. (F) Morphological images of implantation sites 3 weeks after OF‐CL/Matrigel plugs were transplanted into immunodeficient mice (*n* = 5 mice for each group). The subcutaneous adipose tissue generated from implantation in the PPP‐treated group harvested on day 21 was significantly smaller than that in the control group following 21‐day growth. (G) Quantification of volume showing the subcutaneous adipose depots generated from OF‐CL/Matrigel plugs in control and PPP groups (^**^ *p* < .01). (H) Representative images of perilipin 1 staining in tissue sections of adipose depots from PPP and control groups (bar = 100 μm). (I) Quantification of adipocyte area of perilipin 1 staining in control and PPP groups (^*^ *p* < .05).

To address the challenge of TED rodent models for drug validation, we carried out a cell line‐based xenograft model in humanised immunodeficient mice by subcutaneously injecting OF‐CL (Figure 4E). PPP reduced 50% of the adipose tissue volumes generated from the OF‐CL implantations after the 21‐day growth period (Figure S5F,G). Immunofluorescence and haematoxylin and eosin staining showed that the PPP group had fewer adipocytes observed and reduced fat vacuole formation in the implantation sites compared with control group (Figures 4H, 4I and S6B,C).

Our study developed an easy‐to‐use and reproducible platform with high‐throughput drug screening, a 3D culture model and an in‐vivo model for TED studies, which was based on a TED patient‐derived OF‐CL with proliferative and adipogenic properties and without severely perturbing genotype inheritance. Through the platform, we found that PPP could suppress adipogenesis by restraining MCE. Future studies can take advantage of this patient‐derived platform to discover drugs for TED and explore PPP outcomes in patients.

## CONFLICT OF INTEREST STATEMENT

The authors declare no conflicts of interest.

## Supporting information

Supporting information
