# Supplementary material for: Repurposing picropodophyllin as a potential thyroid eye disease treatment via delaying mitotic clonal expansion through a patient‐derived preclinical platform
Source: Clin Transl Med. 2023 Apr 3;13(4):e1218. doi: 10.1002/ctm2.1218 (PMC10070929; doi:10.1002/ctm2.1218)
Supplement: Supplementary file 1 — Supporting information [file CTM2-13-e1218-s001.docx]

Letter-to-Editor with previous submission number: CTM2-2022-09-1486

**Repurposing picropodophyllin as a potential thyroid eye disease treatment via delaying mitotic clonal expansion through a patient-derived preclinical platform**

Dear Editor,

Thyroid eye disease (TED), the most frequent orbital disease and the leading cause of proptosis in adults, is a debilitating, disfiguring, and even sight-threatening disease.^1^ Drugs as alternative options are required for TED patients who need surgery to control orbital compression. Targeting the enhanced adipogenesis of orbital tissue removed by surgery has therapeutic potential for TED by inhibiting adipogenesis of orbital fibroblasts (OFs).^2^ However, translational research is hampered by the limited number of OFs and the lack of reproducible preclinical platforms for drug discovery.^3^ Thus, we generated a TED patient-derived orbital fibroblast cell line (OF-CL) with proliferative and adipogenic capacity, which was further applied in high-throughput drug screening systems, 2D and 3D in vitro models, and xenograft in vivo models for drug-repurposing in TED without a dramatic loss of inherited genotypes. Taking advantage of drug repurposing, we identified Picropodophyllin (PPP), recognized as an inhibitor of IGF1-R and in clinical phase II in lung cancer, with anti-adipogenic therapeutic potentials for TED.

The OF-CL was established through immortalization of OFs which were isolated from the orbital adipose tissue of TED patient (Figure S1 and 1A). It possessed a fibroblast-like morphology and the enhanced proliferative capacity (Figure 1B and C). RNA-sequencing analysis indicated the proliferation-characteristics at transcriptomic levels (Figure 1D and E, Figure S2A and B). We also evaluated the adipogenesis capacity of OF-CL and OFs (Figure 1F). Brightfield images and bodipy staining showed similar formations of intracellular lipid droplets during adipogenic differentiation (Figure 1G and H, Figure S2C), which were confirmed by RNA-sequencing analysis and qPCR test with genes involved in the formation of mature adipocytes and lipid synthesis including (*C/EBP*)*α,* Fatty acid binding protein 4 (*FABP4*), Perilipin 1 (*PLIN1*), Peroxisome proliferator activated receptor gamma (*PPARγ) and* Adiponectin (*ADIPOQ*) (Figure S2D-F, Figure S3).^2^

Drug repurposing is a safe and successful strategy to speed up the drug discovery and development processes upon existing drugs.^4^ To identify candidates for drug repurposing in TED adipogenesis upon patient-derived OF-CL, we hand-curated a collection of 153 small molecule compounds targeting genes involved in adipogenesis pathways of white adipose tissue (WAT) from registered clinical trials firstly (Table S1).^5^ To speed up the screening, we set up a quantitative high throughput screening (qHTS) system based on patient-derived OF-CL to test the rate of anti-adipogenesis of these 153 compounds via bodipy staining (Figure 2A). As shown in Figure 2B, although there were no beneficial effects on anti-adipogenesis of TED by several compounds, which have been documented to inhibit adipogenesis in mesenteric adipose tissue, such as Resveratrol and Genistein. We identified 25 candidates with anti-adipogenic therapeutic potential for TED (Table S1). The main documented targets of these compounds include VEGFR, c-Kit, IGF-1R, mTOR, p38 MAPK, COX and so on.

Picropodophyllin (PPP) among 25 candidates is in first and second phase clinical trials of lung cancer,^6^ and shares the IGF1R target with Teprotumumab antibody which is the first and only FDA-approved drug of TED.^7^ To validate the in vitro inhibitory effect of PPP on adipogenesis of TED, we investigated the anti-adipogenesis effect of PPP on primary OFs. It showed a dose-dependent decrease of intracellular lipid accumulation and adipogenesis-related mRNA expression (Figure 2C-E).

To explore the anti-adipogenesis mechanisms of PPP, we investigated the adipogenesis-stages disturbed by PPP (Figure 3A).^8^ PPP suppressed expression of *C/EBPδ* and *C/EBPβ* in the early stage, C/EBPα and PPARγ in the intermediate stage and FABP4 and ADIPOQ in the late stage (Figure 3B and Figure S4). During early stage of differentiation, preadipocytes were reported to undergo mitotic clonal expansion (MCE) with about two rounds of division to proliferation.^9^ *C/EBPδ* and *C/EBPβ* are the core transcription factors of MCE. To investigate whether PPP affects the early proliferation, we observed the reduction of cell number in PPP-treated group at 48h and 96h (Figure 3C), and decrease of proliferation indicated by EdU positive cells (Figure 3D and E). The MCE regulatory genes including cyclin dependent kinase (*CDK*)4, *CDK6* and *Cyclin D1* in the first 24h of adipogenesis were also suppressed by PPP at transcriptional levels (Figure S5A).

To further determine the specific phases of the cell cycle blocked by PPP during MCE, ploidy analysis was performed to analyze the cell distribution at 24h and 16h after differentiation initiation. Phosphorylate Histone H3, a M phases cell marker, was used to distinguish cells in G2 phase or M phase (Figure 3F-I, Figure S5B-E). These data suggest that PPP may cause G2/M arresting and inhibit the MCE process (Figure 3J, Figure S5F).

3D culture models provide an alternative to 2D cell culture to closely mimic the cellular microenvironment in diseases.^10^ To assess the anti-adipogenesis function in 3D-culture systems, we employed sodium carboxymethyl cellulose and Matrigel to form a fibroblast spheroid (Figure 4A and B). After 21-day adipogenic differentiation, PPP suppressed 68.31% lipid accumulation in spheroids of OFs compared with control (Figure 4C and D). The mRNA levels of adipogenic marker genes were significantly reduced in the PPP group (Figure S6A).

To address the challenge of TED rodent models for drug validation, we carried out a cell line-based xenograft model in humanized immunodeficient mice by subcutaneously injecting OF-CL (Figure 4E). PPP reduced 50% of the adipose tissue volumes generated from the OF-CL implantations after the 21-day growth period (Figure 4F and G). Immunofluorescence and H&E staining showed that PPP group had less adipocytes observed and reduced fat vacuole formation in the implantation sites compared with control group (Figure 4H and 7I, Figure S6B and C).

Our study developed an easy-to-use and reproducible platform with high-throughput drug screening, a 3D culture models and a in vivo model for TED studies, which was based on a TED patient-derived OF-CL with proliferative and adipogenic properties and without severely perturbing genotype inheritance. Through the platform, we find that PPP could suppress adipogenesis via restraining mitotic clonal expansion. Future studies can take advantage of this patient-derived platform to discovery drug for TED, and explore PPP outcomes in patients (Figure S7).

# ACKNOWLEDGEMENT

This work was supported by the National Natural Science Foundation of China (32171177, 92057106, 82171076), Natural Science Foundation of Shanghai (19ZR1440500), Shanghai Hospital Development Center (SHDC2020CR2040B), Shanghai Collaborative Innovation Center for Transnational Medicine (TM202115PT), Shanghai Pujiang Program (2019PJD046) and Shanghai Jiaotong University-Gaofeng Clinical Medicine Grant Support.

# CONFLICT OF INTEREST

The authors declare that they have no conflict of interest.

# AUTHOR CONTRIBUTIONS

FZ conceptualized the project. FZ and JH designed the experiments. JH, QHJ, JLW, LZ, NS and LCS conducted the experiments. JH, GYH, KJL and QZJ analyzed the data. JH, DSP, DSJ, YYS, LC and JZ contributed to the animal experiments; JH wrote the manuscript. FZ, HC, and XDS revised the manuscript. All authors read and approved the final manuscript.

# DATA AVAILABILITY STATEMENT

The data that support the findings of this study are available on request from the corresponding author, FZ，upon reasonable request.

*Jing Hu* ^1,2^

*Jiali Wu* ^1,2^

*Qihuang Jin* ^1,2^

*Ling Zhang* ^1,2^

*Ning Shen* ^1,2^

*Yiyang Shu* ^1,2^

*Lu Cheng* ^1,2^

*Jian Zhang* ^1,2^

*Guangyi Hu* ^1,2^

*Kangjia Lv* ^1,2^

*Qizhi Jian* ^1,2^

*Hui Chen* ^1,2,3,4,5,6,7^*

*Fang Zhang* ^1,2,3,4^*

*Xiaodong Sun* ^1,2,3,4^*

^1^ National Clinical Research Center for Eye Diseases, Shanghai General Hospital, Shanghai Jiao Tong University School of Medicine, Shanghai, China

^2^ Shanghai Key Laboratory of Fundus Diseases, Shanghai, China

^3^ Shanghai Engineering Center for Visual Science and Photomedicine, Shanghai, China

^4^ Shanghai Engineering Center for Precise Diagnosis and Treatment of Eye Diseases, Shanghai, China

^5^ School of Medicine, University of Electronic Science and Technology of China,

Chengdu, Sichuan Province, China.

^6^ Eye School of Chengdu University of Traditional Chinese Medicine,

Chengdu, Sichuan Province, China.

^7^ University of Shanghai for Science and Technology, Shanghai, China.

**Correspondence**

Fang Zhang, Hui Chen and Xiaodong Sun, National Clinical Research Center for Eye Diseases, Shanghai General Hospital, Shanghai Jiao Tong University School of Medicine,

Shanghai 200080, China

Email: zhangfang2018@sjtu.edu.cn; 352477354@qq.com; drxuxun@sjtu.edu.cn;

# REFERENCES

1. Davies, T.F., et al., Graves' disease. Nat Rev Dis Primers, 2020. 6(1): p. 52.
2. Cheng, L., et al., Repurposing Lenvatinib as A Potential Therapeutic Agent against Thyroid Eye Disease by Suppressing Adipogenesis in Orbital Adipose Tissues. Pharmaceuticals (Basel), 2022. 15(11).
3. Bartalena, L., et al., The 2021 European Group on Graves' orbitopathy (EUGOGO) clinical practice guidelines for the medical management of Graves' orbitopathy. Eur J Endocrinol, 2021. 185(4): p. G43-G67.
4. Talevi, A. and C.L. Bellera, Challenges and opportunities with drug repurposing: finding strategies to find alternative uses of therapeutics. Expert Opin Drug Discov, 2020. 15(4): p. 397-401.
5. Jiang, Y., A.Y. Jo, and J.M. Graff, SnapShot: adipocyte life cycle. Cell, 2012. 150(1): p. 234-234 e2.
6. Aiken, R., et al., Phase I clinical trial of AXL1717 for treatment of relapsed malignant astrocytomas: analysis of dose and response. Oncotarget, 2017. 8(46): p. 81501-81510.
7. Smith, T.J., et al., Teprotumumab for Thyroid-Associated Ophthalmopathy. N Engl J Med, 2017. 376(18): p. 1748-1761.
8. Farmer, S.R., Transcriptional control of adipocyte formation. Cell Metab, 2006. 4(4): p. 263-73.
9. Tang, Q.Q. and M.D. Lane, Adipogenesis: from stem cell to adipocyte. Annu Rev Biochem, 2012. 81: p. 715-36.
10. Pampaloni, F., E.G. Reynaud, and E.H. Stelzer, The third dimension bridges the gap between cell culture and live tissue. Nat Rev Mol Cell Biol, 2007. 8(10): p. 839-45.

# FIGURE

**FIGURE 1** Generation and assessment of a human orbital fibroblast cell line (OF-CL) from a TED patient. (A) Schematic view of the construction of an orbital adipose tissue (OAT) derived OF-CL from a TED patient. Stromal vascular fraction (SVF) was isolated from an OAT of a 35-year-old female TED patient who underwent decompression surgery, and cultured in dish for primary orbital fibroblasts (OFs). SV40 large Tantigen was transformed in OFs using retroviral system to generate the immortalized OF-CL. (B) brightfield microscopy photo of OFs and OF-CL (Bar= 200μm). (C) Doubling time of different generations of OFs and OF-CL (*P<0.05, **P<0.01). (D) Volcano plot showed differentially expressed genes (DEGs) between OFs and OF-CL with absolute Fold Change ≥ 2 and FDR< 0.05 (n= 3 for each cell type). Blue and red dots indicated up-regulated DEGs in OF and OF-CL groups respectively. All other genes were labeled as gray dots. (E) Gene ontology (GO) analysis of DEGs showed that proliferation-related signaling pathways were enriched in OF-CL. (F) Schematic timeline of adipogenic differentiation and assessment in the 2D culture of OFs or OF-CL. 8 x10^3^ cells were plated into each well of 96-well plates, and grew to 100% confluence in growth medium on Day -3. Cells were cultured for another three days in normal growth conditions before induction medium was employed on Day 0, and the medium was refreshed every 3 days. The medium was changed into the maintenance medium on Day 6 and refreshed every three days. RNA extraction was conducted on the Day 6 for detection of adipogenic differentiation marker genes. An assessment of lipid accumulation was performed on Day 14. (G) Bodipy and DAPI staining indicated the intracellular lipid droplets of adipocytes on Day 14 (Bar= 50μm). (H) The quantitative analysis of relative Bodipy intensity (***P<0.001).

**FIGURE 2** Picropodophyllin (PPP) isolated from high-throughput screening shows anti-adipogenesis potentials to TED upon OF-CL and OFs differentiation. (A) Strategy of the high-throughput screening for small molecules with anti-adipogenesis potential based on patient-derived OF-CL. After 14 days of adipogenic differentiation treated with each compound at indicated concentration, Bodipy and DAPI staining were conducted to quantitatively evaluate the amount of lipid accumulation in each well. (B) Scatter plot of inhibition rates of adipogenesis was calculated through Bodipy intensity for each compound assessed in the high-throughput screening upon OF-CL differentiation. Green and purple dots representing 1μM and 10μM concentration groups respectively (n= 2 for each treatment). (C) With the increase of PPP concentration, the relative Bodipy intensity of differentiated OFs decreased on Day 14 after adipogenesis induction (Bar= 50μm). (D) The quantitative analysis of relative Bodipy intensity was shown (###P<0.001 verse undifferentiated control, ***P<0.001 verse differentiated control). (E) The mRNA expression level of adipogenesis marker genes were detected by RT–qPCR analysis (***P<0.001 verse differentiated control).

**FIGURE 3** PPP inhibits adipogenesis by affecting the mitotic clonal expansion in early differentiation. (A) Schematic representation of different stages during adipogenic differentiation. (B) RT–qPCR analysis of C/EBPβand C/EBPδafter adipogenic induction with DMSO or 1μM PPP treatment (**P<0.01, ***P<0.001). (C) Cell numbers of 0, 4, 18, 24, 48, and 96 hours after adipogenic induction treated with DMSO or 1μM PPP (***P<0.001). (D) Representative images of EdU staining in differentiated OF-CL treated with DMSO or 1μM PPP for 48h (Bar= 50μm). (E) Quantification of the ratio of EdU positive cells to total Hoechst positive cells (*P<0.05). (F) Flow cytometry analysis of cell cycle distribution in DMSO-treated and PPP-treated group at 24h after adipogenic differentiation. Cell cycle distribution was determined by flow cytometry using propidium iodide staining. (G) The percentages of orbital fibroblast cell line (OF-CL) treated with induction medium supplemented with 1μM PPP or DMSO for 24h at different cell cycle phases were analyzed by ModFit software (*P<0.05, ***P<0.001). (H) Flow cytometry analysis of p-Histone H3 positive cells in DMSO-treated and PPP-treated group at 16h or 24h after adipogenic differentiation. (I) The percentages of p-Histone H3 positive cells treated with induction medium supplemented with 1μM PPP or DMSO for 24h were analyzed by FlowJo software (**P<0.01, ***P<0.001). (J) Percentage of cells in G0/G1, S, G2, and M phases after treatment with DMSO or PPP.

**FIGURE 4** PPP attenuates adipogenesis in 3D cell culture model and mammalian fat depots of immunodeficient mice implantation model. (A) Schematic depicting the construction of 3D cell culture model formed by OFs with Matrigel. 2 × 10^6^ cells were resuspended in 1ml growth medium and a medium drop of 25μl was placed under the lid for spheroid formation. After being transferred into Matrigel and followed by adipogenic differentiation, spheroids were collected and evaluated on Day 14. (B) Brightfield images of OFs 3D spheroids on Day -3, 0, and 3 (Bar= 200μm). (C) Representative images of DAPI and Bodipy staining 3D spheroids on Day 14 (Bar= 250μm). (D) Quantitative analysis of the relative Bodipy fluorescence intensity in 3D spheroids of two groups (**P<0.01). (E) Schematic depicting the construction of OF-CL adipogenesis model in vivo. Total 2*10^6^ OF-CL pre-treated with induction medium supplemented with DMSO or PPP for 24h were implanted into the left and right inguinal area of immunodeficient mice respectively. Mice were sacrificed on Day 21 and the OF-CL/Matrigel plugs were harvested. (F) Morphological images of implantation sites three weeks after OF-CL/Matrigel plugs were transplanted in immunodeficient mice (n= 5 mice for each group). The subcutaneous adipose tissue generated from implantation in the PPP-treated group harvested on Day 21 was significantly smaller than that in the control group following 21-day growth. (G) Quantification of volume showing the subcutaneous adipose depots generated from OF-CL/Matrigel plugs in control and PPP groups (**P<0.01). (H) Representative images of perilipin 1 staining in tissue sections of adipose depots from PPP and control groups (Bar = 100μm). (I) Quantification of adipocyte area of perilipin 1 staining in control and PPP groups (*P<0.05).

# SUPPLEMENTARY

**FIGURE S1 Computed tomography image and H&E-stained sections of orbital adipose tissue in TED patients.** (A) The computed tomography image showed the enhancement of the orbital adipose tissue (white arrow) in the thyroid eye disease patient. (B) H&E stained section of orbital adipose tissue of thyroid eye disease demonstrated adipose tissue morphology (Bar = 100μm).

**FIGURE S2** Assessment of OF-CL proliferation and adipogenic differentiation capacity. (A) Gene set enrichment analysis (GSEA) profiles indicated a significant enrichment of gene sets associated with DNA replication and DNA strand elongation. (B) Heatmap of RNA-seq data presented the proliferation-related DEGs. (C) The brightfield photographs illustrate the morphology of intracellular lipid droplets during the differentiation process from fibroblasts into mature adipocytes on Day 0, 3, 6, and 9 respectively, and the representative pictures of Oil-Red-O staining adipocytes on Day 14 (Bar= 50μm). (D, E) Volcano plot of the DEGs between undifferentiated and differentiated cells which OFs shown in the upper panel and OF-CL shown in the lower panel. (F) RT–qPCR analysis indicated the expression of adipogenic marker genes in OFs and OF-CL between undifferentiated and differentiated cells (***P<0.001).

**FIGURE S3** Differentiated OF-CL and OFs show similar performance on adipogenesis-related marker genes and pathways. (A) Heatmap of adipogenesis marker gene between undifferentiated and differentiated OFs or OF-CL. (B) Gene Set Enrichment Analysis (GSEA) of DEGs between undifferentiated and differentiated cells showed similar enrichment patterns in adipocytokine signaling pathway, PPAR signaling pathway, and fatty acid metabolic process.

**FIGURE S4** PPP inhibits adipogenesis by downregulated C/EBPα、PPARγ2、FABP4 and ADIPOQ at the late stage of adipogenesis. RT–qPCR analysis of adipogenesis gene expression at different times after adipogenic induction with DMSO or 1μM PPP treatment (**P<0.01, ***P<0.001).

**FIGURE S5** PPP inhibits the mitotic clonal expansion by downregulating CDK4, CDK6 and cyclin D1 and cell cycle arresting. (A) RT–qPCR analysis of marker genes of mitotic clonal expansion including CDK2, CDK4 and CDK6 (*P<0.05, **P<0.01, ***P<0.001). (B) Flow cytometry analysis of cell cycle distribution in DMSO-treated and PPP-treated group at 16h after adipogenic differentiation. (C) The percentages of orbital fibroblast cell line (OF-CL) treated with induction medium supplemented with 1μM PPP or DMSO for 16h at different cell cycle phases (*P<0.05, ***P<0.001). (D) Flow cytometry analysis of p-Histone H3 positive cells in DMSO-treated and PPP-treated group at 16h after adipogenic differentiation. (E) The percentages of p-Histone H3 positive cells treated with induction medium supplemented with 1μM PPP or DMSO for 16h (**P<0.01, ***P<0.001). (I) Percentage of cells in G0/G1, S, G2, and M phases at 16h.

**FIGURE S6** PPP suppresses adipogenesis in the 3D cell culture model and in vivo model. (A) RT–qPCR analysis of marker genes of adipogenesis in 3D spheroids of control or PPP group (**P<0.01). (B) Representative images of sections of adipose tissue of control and PPP group after H&E staining (Bar= 100μm). (C) The percentage of adipocyte area induced by implantation was lower in the PPP group than that in the control group (*P<0.05).

**FIGURE S7** Graphical abstract. (A) We generated a renewable patient-derived orbital fibroblast cell line (OF-CL) for drug discovery and mechanism investigation of thyroid eye diseases (TED). (B, C) Based on this cell line, an easy-to-use and reproducible platform with high-throughput screening systems, 2D and 3D cell culture models, and xenograft mouse models for anti-adipogenesis studies was established for drug repurposing approach, which identified 25 candidate small molecules. (D) Picropodophyllin, an inhibitor of IGF1-R in Phase II clinical trials of lung cancer, can reduced adipogenesis in TED models through delaying mitotic clonal expansion via CEBPβ and CEBPδ reduction and cell cycle arresting.

**SUPPLEMENTARY TABLE S1**

List of Compounds used for qHTS.

| Catalog NO. | Drug Name | Target |
| --- | --- | --- |
| S1005 | Axitinib | c-Kit, PDGFR, VEGFR |
| S1010 | Nintedanib (BIBF 1120) | FGFR, PDGFR, VEGFR |
| S1022 | Ridaforolimus (Deforolimus) | mTOR |
| S1029 | Lenalidomide (CC-5013) | TNF-alpha |
| S1035 | Pazopanib HCl (GW786034 HCl) | c-Kit, PDGFR, VEGFR |
| S1039 | Rapamycin (Sirolimus) | Autophagy, mTOR |
| S1040 | Sorafenib Tosylate | PDGFR, Raf, VEGFR |
| S1042 | Sunitinib Malate | c-Kit, PDGFR, VEGFR |
| S1044 | Temsirolimus (CCI-779) | mTOR |
| S1082 | Vismodegib (GDC-0449) | Hedgehog/Smoothened |
| S1119 | Cabozantinib (XL184) | c-Met, VEGFR |
| S1120 | Everolimus (RAD001) | mTOR |
| S1164 | Lenvatinib (E7080) | VEGFR |
| S1178 | Regorafenib (BAY 73-4506) | c-RET, VEGFR |
| S1193 | Thalidomide | E3 Ligase, TNF-alpha |
| S1197 | Finasteride | 5-alpha Reductase |
| S1202 | Dutasteride | 5-alpha Reductase |
| S1206 | Bisoprolol fumarate | Adrenergic Receptor |
| S1207 | Tivozanib (AV-951) | c-Kit, PDGFR, VEGFR |
| S1255 | Nepafenac | COX |
| S1261 | Celecoxib | COX |
| S1342 | Genistein | EGFR, Topoisomerase |
| S1396 | Resveratrol | Autophagy |
| S1443 | Zileuton | Lipoxygenase |
| S1458 | VX-745 | p38 MAPK |
| S1467 | Doxercalciferol | Vitamin |
| S1468 | Alfacalcidol | Vitamin |
| S1490 | Ponatinib (AP24534) | Bcr-Abl, FGFR, PDGFR, VEGFR |
| S1491 | Fludarabine | DNA/RNA Synthesis, STAT |
| S1500 | Betamethasone | Glucocorticoid Receptor |
| S1547 | Febuxostat | ROS |
| S1548 | Dapagliflozin | SGLT |
| S1549 | Nebivolol HCl | Adrenergic Receptor |
| S1567 | Pomalidomide | TNF-alpha |
| S1614 | Riluzole | GluR, Sodium Channel |
| S1622 | Prednisone | Glucocorticoid Receptor |
| S1623 | Acetylcysteine | TNF-alpha, ROS |
| S1628 | Triamcinolone Acetonide | Glucocorticoid Receptor |
| S1629 | Orlistat | Lipase, Fatty Acid Synthase |
| S1669 | Loteprednol etabonate | Glucocorticoid Receptor |
| S1680 | Disulfiram | Dehydrogenase |
| S1681 | Mesalamine | IκB/IKK |
| S1688 | Betamethasone Dipropionate | Glucocorticoid Receptor |
| S1689 | Meprednisone | Glucocorticoid Receptor |
| S1690 | Betamethasone Valerate | Glucocorticoid Receptor |
| S1696 | Hydrocortisone | Glucocorticoid Receptor |
| S1701 | Desonide | Glucocorticoid Receptor |
| S1729 | Gemfibrozil | PPAR |
| S1733 | Methylprednisolone | Glucocorticoid Receptor |
| S1734 | Meloxicam | COX |
| S1737 | Prednisolone | Glucocorticoid Receptor |
| S1759 | Pitavastatin Calcium | HMG-CoA Reductase |
| S1792 | Simvastatin | HMG-CoA Reductase |
| S1794 | Fenofibrate | PPAR |
| S1827 | Betaxolol HCl | Adrenergic Receptor |
| S1848 | Curcumin | Nrf2, NF-κB, HDAC |
| S1856 | Metoprolol Tartrate | Adrenergic Receptor |
| S1888 | Deflazacort | Glucocorticoid Receptor |
| S1899 | Nicotinamide (Vitamin B3) | Sirtuin |
| S1909 | Fluvastatin Sodium | HMG-CoA Reductase |
| S1921 | Phenindione | Vitamin |
| S1933 | Triamcinolone | Glucocorticoid Receptor |
| S1937 | Isoniazid | Fatty Acid Synthase |
| S1950 | Metformin HCl | Autophagy |
| S1987 | Mometasone furoate | Glucocorticoid Receptor |
| S1992 | Fluticasone propionate | Glucocorticoid Receptor |
| S2015 | Suplatast Tosylate | IL Receptor |
| S2046 | Pioglitazone HCl | P450 (e.g., CYP17) |
| S2061 | Lovastatin | HMG-CoA Reductase |
| S2075 | Rosiglitazone HCl | PPAR |
| S2077 | Atorvastatin Calcium | HMG-CoA Reductase |
| S2091 | Betaxolol | Adrenergic Receptor |
| S2151 | Sonidegib (Erismodegib) | Hedgehog/Smoothened |
| S2169 | Rosuvastatin Calcium | HMG-CoA Reductase |
| S2226 | Idelalisib (CAL-101, GS-1101) | PI3K |
| S2261 | Andrographolide | NF-κB |
| S2264 | Artemether | Anti-infection |
| S2265 | Artesunate | STAT |
| S2289 | Daidzin | Dehydrogenase |
| S2362 | Synephrine | Adrenergic Receptor |
| S2390 | Polydatin | Phospholipase (e.g. PLA) |
| S2391 | Quercetin | Src, Sirtuin, PKC, PI3K |
| S2470 | Fluocinolone Acetonide | Glucocorticoid Receptor |
| S2505 | Rosiglitazone maleate | PPAR |
| S2522 | L-Adrenaline | Adrenergic Receptor |
| S2542 | Phenformin HCl | AMPK |
| S2554 | Daphnetin | PKA, EGFR, PKC |
| S2559 | Cortisone acetate | Glucocorticoid Receptor |
| S2566 | Isoprenaline HCl | Adrenergic Receptor |
| S2570 | Prednisolone Acetate | Glucocorticoid Receptor |
| S2584 | Clobetasol propionate | Glucocorticoid Receptor |
| S2590 | Pioglitazone | PPAR |
| S2604 | Dehydroepiandrosterone (DHEA) | Estrogen/progestogen Receptor |
| S2608 | Fluocinonide | Glucocorticoid Receptor |
| S2664 | Clinofibrate | HMG-CoA Reductase |
| S2665 | Ciprofibrate | PPAR |
| S2907 | Pirfenidone | TGF-beta/Smad |
| S3012 | Pazopanib | c-Kit, PDGFR, VEGFR |
| S3017 | Aspirin | COX |
| S3036 | Pravastatin sodium | HMG-CoA Reductase |
| S3130 | Biotin (Vitamin B7) | Vitamin |
| S3604 | Triptolide (PG490) | NF-κB |
| S3612 | Rosmarinic acid | IκB/IKK |
| S3648 | Amlexanox | Immunology & Inflammation related |
| S4001 | Cabozantinib malate (XL184) | TAM Receptor, VEGFR |
| S4010 | Acebutolol HCl | Adrenergic Receptor |
| S4085 | Levobetaxolol HCl | Adrenergic Receptor |
| S4088 | Flumethasone | Glucocorticoid Receptor |
| S4123 | Timolol Maleate | Adrenergic Receptor |
| S4159 | Bezafibrate | PPAR |
| S4171 | Choline Chloride | AChR |
| S4223 | Mevastatin | HMG-CoA Reductase |
| S4238 | Cepharanthine | TNF-alpha |
| S4267 | Diacerein | IL Receptor |
| S4268 | Flufenamic acid | Immunology & Inflammation related |
| S4278 | Carteolol HCl | Adrenergic Receptor |
| S4527 | Fenofibric acid | PPAR |
| S4580 | Hydroquinone | ROS |
| S4609 | Diflunisal | COX |
| S4817 | Atenolol | Adrenergic Receptor |
| S5121 | Phytol | PPAR |
| S5234 | Nintedanib Ethanesulfonate Salt | VEGFR, FGFR, PDGFR |
| S5240 | lenvatinib Mesylate | VEGFR |
| S5248 | Apatinib | VEGFR, c-RET |
| S5360 | Diflorasone | Glucocorticoid receptor |
| S5413 | Ertugliflozin | SGLT2 |
| S5430 | Metoprolol | Adrenergic Receptor |
| S5486 | Fluorometholone | Glucocorticoid Receptor |
| S5501 | Hydrocortisone acetate | Glucocorticoid receptor |
| S5667 | Fruquintinib | VEGFRs |
| S5685 | Desoximetasone | Glucocorticoid Recep |
| S5713 | Pravastatin | HMG-CoA Reductase |
| S5715 | atorvastatin | HMG-CoA Reductase |
| S5734 | Midodrine hydrochloride | Adrenergic Receptor |
| S5735 | Midodrine | Adrenergic Receptor |
| S5739 | Nebivolol | Adrenergic Receptor |
| S5812 | Choline Fenofibrate | PPAR |
| S7083 | Ceritinib (LDK378) | ALK |
| S7091 | Zotarolimus(ABT-578) | mTOR |
| S7668 | Picropodophyllin (PPP) | IGF-1R |
| S7781 | Sunitinib | PDGFR, c-Kit, VEGFR |
| S7954 | CP21R7 (CP21) | Wnt/beta-catenin |
| S8034 | Apremilast (CC-10004) | TNF-alpha, PDE |
| S8432 | Troglitazone (CS-045) | PPAR |
| S8558 | Tofogliflozin(CSG 452) | SGLT |
| S9507 | (−)-Norepinephrine | Adrenergic Receptor |
| S9015 | Homoharringtonine | STAT |
| S4028 | Dexamethasone Sodium Phosphate | IL Receptor, Autophagy, COX |
| S1046 | Vandetanib (ZD6474) | VEGFR |
| S2251 | (-)-Huperzine A (HupA) | GluR, AChR |
| S4218 | Amoxapine | GlyT |
| S5601 | Balofloxacin Dihydrate | PPARD |
